# Supplementary material for: Myelin Oligodendrocyte Glycoprotein (MOG)35–55 Mannan Conjugate Induces Human T-Cell Tolerance and Can Be Used as a Personalized Therapy for Multiple Sclerosis
Source: Int J Mol Sci. 2024 May 31;25(11):6092. doi: 10.3390/ijms25116092 (PMC11172913; doi:10.3390/ijms25116092)
Supplement: Supplementary file 1 [file ijms-25-06092-s001.zip › ijms-3004121-supplementary.pdf]

Myelin oligodendrocyte glycoprotein (MOG)35-55 mannan conjugate induces human T-cell tolerance and can be used as a personalized therapy for multiple sclerosis

Maria Rodi, Anne-Lise de Lastic, Ioannis Panagoulias, Ioanna Aggeletopoulou, Kostas Kelaidonis, John Matsoukas, Vasso Apostolopoulos, Athanasia Mouzaki

Supplementary Figure S1

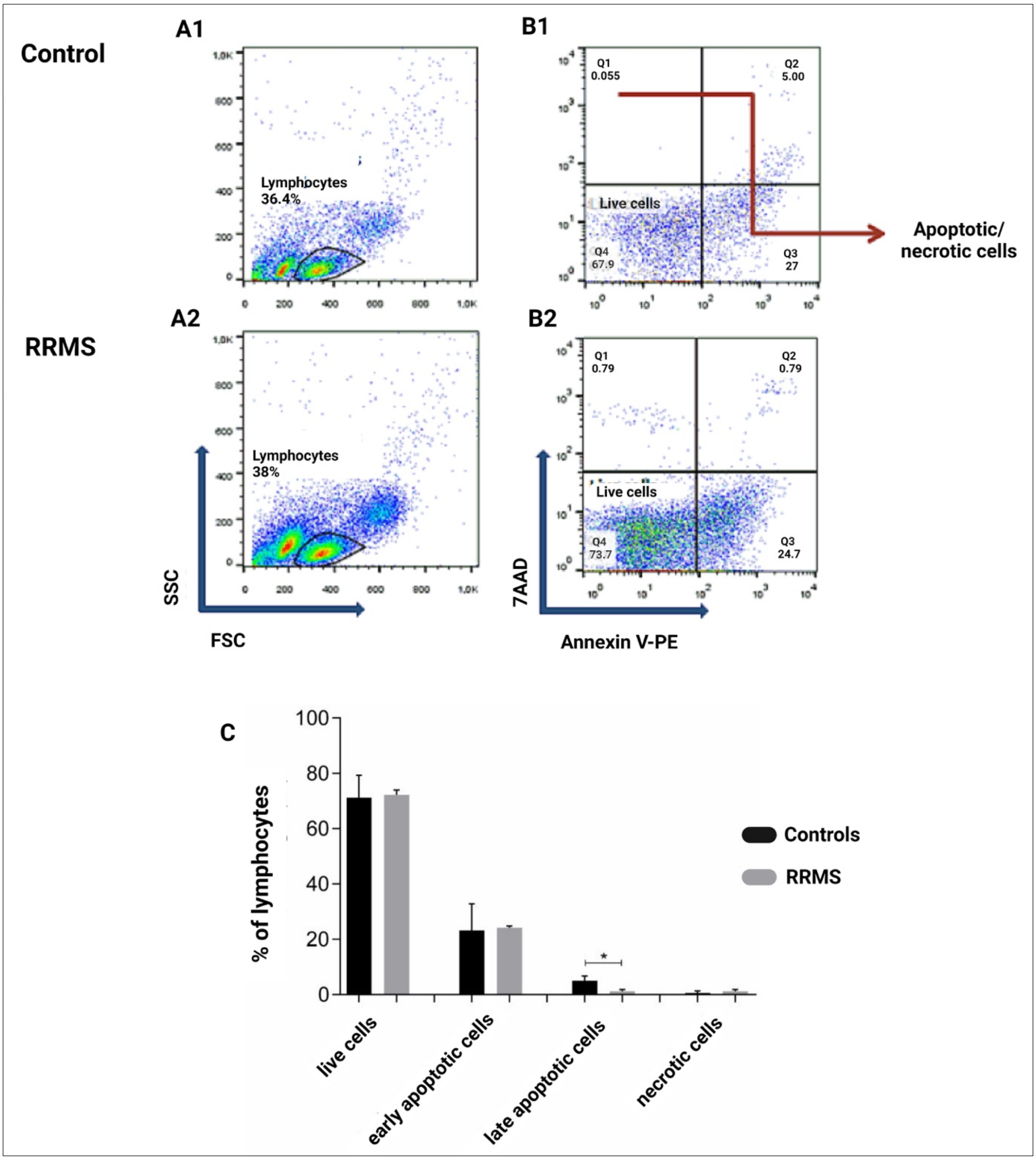

Figure S1. Estimation of lymphocyte viability in stored PBMCs before addition to DC cultures by flow cytometry. **A1, B1:** Representative analysis of a control sample; **A2, B2:** Representative analysis of a patient sample; **C:** Collective data. The asterisk indicates a statistically significant difference between late apoptotic lymphocyte levels in PBMCs from patients and controls (*$p<0.05$).
